# Is Syphilis Hereditary?

**Published:** 1915-02

**Authors:** 


**Is Syphilis Hereditary?** E. Kilbourne Tullidge, New York, "Detroit Med. Jour.," December, 1914. The majority of modern biologists disbelieve in the transmissibility of acquired characteristics in general, which include disease and deformity. Mammalian ova have not been shown to be phagocytic and Gartner has definitely shown that spermatozoa are not. Rohlff and Gartner have calculated the number of spermatozoa in an ejaculation at over 26,000,000. (Note: The editor reported in the "N. Y. Med. Jour.," June 4, 1910, three counts by haemocytometer, respectively 346 million, 2,672 million and 286 million). Owing, possibly to misprints, we are unable to follow the mathematic argument as to the probability of true hereditary infection of the ovum but it is extremely small. Colles' law as to the possibility of non-infection of the mother of a syphilitic child is no longer held to be true. Various experiments are cited as to the possibility of infection through the placenta, hence locating primarily in the liver of the foetus, according with actual observations. Even if syphilis were hereditary, the mother would also be infected from the foetus.

